# Novel *Mycoplasma bovis* membrane lipoproteins induce the inflammatory response of host epithelial cells and macrophage

**DOI:** 10.3389/fimmu.2025.1580436

**Published:** 2025-06-09

**Authors:** Xiaotan Yuan, Jiating Ma, Yiming Wu, Gang Zhao, Yujiong Wang

**Affiliations:** ^1^ Key Laboratory of Ministry of Education for Conservation and Utilization of Special Biological Resources in the Western China, Ningxia University, Yinchuan, China; ^2^ School of Life Sciences, Ningxia University, Yinchuan, China

**Keywords:** immunogenicity, inflammatory response, membrane lipoproteins, *Mycoplasma bovis*, secreted lipoproteins

## Abstract

**Background:**

Mycoplasmopsis bovis, caused by *Mycoplasma bovis* infection, can lead to severe pneumonia and mastitis in cattle, resulting to significant economic losses to the global cattle industry. The membrane lipoprotein of *M. bovis* is recognized as a critical virulence factor, playing a key role in pathogenesis and modulating host cell immune responses. Therefore, identifying *M. bovis* membrane lipoproteins is of great significance to explore their roles in regulating the immune response of host cells.

**Methods:**

In this study, 10 immunogenic *M. bovis* membrane lipoproteins were predicted by bioinformatics software, with 8 of them subsequently expressed and purified. Four lipoproteins, namely MbovP0659, MbovP0536, MbovP0393 and MbovP0585 were then shown to react with *M. bovis*-infected cattle serum, hence demonstrated their significantly stronger immunogenicity compared with others. Furthermore, polyclonal antibodies against the eight membrane lipoproteins were prepared and used to detect their subcellular localization.

**Results:**

The results revealed that MbovP0592 was a secreted lipoprotein, while MbovP0659, MbovP0536, MbovP0393 and MbovP0585 were confirmed as membrane lipoproteins. Finally, the effects of MbovP0592, MbovP0659, MbovP0536, MbovP0393 and MbovP0585 on the inflammatory cytokines of host epithelial cells and macrophages were characterized by qRT-PCR.

**Conclusion:**

This study identified a secretory lipoprotein MbovP0592 along with four immunogenic membrane lipoproteins, all of which possess the ability to initiate inflammatory responses in host cells. These results provide potential candidates for developing subunit vaccines and study the pathogenic mechanism of *M. bovis*.

## 1 Introduction

*Mycoplasma bovis* (*M. bovis*), the smallest microorganism without a cell wall and capable of self-replication *in vitro*, predominantly affects cattle, causing symptoms such as pneumonia, mastitis, arthritis, otitis media, conjunctivitis and meningitis, just to name a few (1). The spread of *M. bovis* has caused significant economic losses to the global cattle breeding industry (2), but due to the organism’s lack of a cell wall, β-lactams and other antibacterial agents targeting cell wall synthesis tend to be ineffective (3). In addition, *M. bovis* lacks classical bacterial virulence factors such as membrane polysaccharides, lysozyme, hemolysin and lectin. Due to these limitations, elucidating the pathogenesis of *M. bovis* has been a major challenge, thus hindering attempts to control and prevent the disease (4).

The increasing availability of whole genome sequences has helped to reveal the genetic basis of *M. bovis*’s pathogenicity (5). However, analyses have shown that this microorganism lacks classical bacterial toxins as well as a complete secretion system, with numerous proteins of unknown functions being instead present (6, 7). In most bacteria, the secretome plays an important role in host interactions, especially in immune evasion and regulation, adhesion, invasion, nutrition acquisition and modulation of host-bacterial interactions (8, 9). At the same time, many bacterial secretome proteins are also involved in virulence, hence making their characterization crucial for developing effective disease control strategies (10). Recent research on the *M. bovis* secretome has shown that its membrane lipoproteins can not only promote adhesion and invasion of host cells but also induce immune responses (11, 12). In particular, membrane lipoproteins with both secretory properties and immunogenicity have become a research focus as they play essential roles in host-pathogen interactions (13). So far, studies have identified the biological functions of some secreted membrane lipoproteins from *M. bovis*: for instance MbovP280 induces Bovine Macrophages (BoMac) cells apoptosis by binding to CRYAB ligands (14), while MbovP475, as a nucleomodulin, binds to the promoters of *MCF2L2* and *CRYAB* genes, down-regulating their expression and reducing BoMac cells’ viability (15). Furthermore, MbovP0145 can induce IL-8 secretion in embryonic bovine lung (EBL) cells by interacting with β-actin (16), while MbovP0725 is a novel secreted serine/threonine phosphatase that can inhibit inflammatory responses and activate both the glycolysis pathway and nucleotide metabolism (17). Altogether, these findings highlight the crucial role of membrane lipoproteins in *M. bovis*’s pathogenicity. However, to date, only a limited number of such lipoproteins have been identified.

Bacterial infections usually trigger inflammatory responses in host cells, releasing cytokines (e.g., TNF-α and interleukins) that affect the cells’ viability and functions (18). Current research on the inflammatory response to *M. bovis* mainly focuses on bacterial-level interactions: *M. bovis* infection activates Toll-like receptors (TLRs) in bovine mammary epithelial (MAC-T) cells, which then initiate downstream MAPK and NF-κB inflammatory signaling pathways, leading to cell damage (19). Additionally, *M. bovis* infection of macrophages (M1-MDMs and M2-MDMs) induces the release of inflammatory cytokines and proteinases(MMP-12 and SPLA2) which exacerbate respiratory symptoms in cattle via inflammatory pathways (20). In contrast to bacterial-level studies, examining the membrane lipoprotein of *M. bovis* provides a more accurate understanding of the microorganism’s virulence at the molecular level. In summary, the inflammatory response, as the host’s first line of defense against bacterial infection, plays a critical role in identifying and removing pathogens. It is, therefore, of great significance for understanding bacterial pathogenic mechanisms and developing effective strategies for preventing and treating infections.

Membrane lipoproteins, which are key components of *M. bovis*, represent important targets for vaccine development. In this study, a novel secretory membrane lipoprotein, MbovP0592, as well as four other membrane lipoproteins with strong immunogenicity were identified, with their roles in inducing inflammatory responses in host cells subsequently confirmed. It is expected that these findings will provide new insights into *M. bovis*’s pathogenesis and suggest potential candidates for developing new vaccines and diagnostic tools.

## 2 Materials and methods

### 2.1 M. bovis strain and cell growth

*M. bovis* strain HB0801 (GenBank accession number: NC _ 018077.1) (21) was cultured in PPLO medium (#255420, BD, USA) for 36 h until the logarithmic phase was reached.

Cultures of bovine mammary epithelial cells, bovine macrophages cells and embryonic bovine lung cells were established in DMEM/F12 medium (#PM150312, Pronosai, China), RPMI-1640 medium (#PM150110, Pronosai, China) and MEM medium (#PM150410, Pronosai, China), respectively prior to incubation in a 5% CO_2_ incubator at 37°C. To each medium, 1% of a penicillin and streptomycin mixture (#15140-122, Gibco, USA) as well as 10% of heat-inactivated FBS (#164210-50, Pronosai, China) were added before use.

### 2.2 SDS-PAGE and western blotting

*M. bovis* was inoculated into 10 mL of PPLO broth at a 1:1000 dilution and cultured to mid-log phase. Bacterial cells were harvested by centrifugation at 14,000 × g for 10 min at room temperature. The supernatant was discarded, and the pellet was resuspended in 500 μL of 1× PBS. A 100 μL aliquot of the resuspended cells was mixed with 6×protein loading buffer(#DL101, transgen biotech, China) and denatured at 100°C for 15 min in a dry bath incubator to prepare whole-cell protein lysates. The protein concentration of *M. bovis* whole-cell lysates was determined using a BCA assay kit(#KGB2101-500, KeyGEN BioTECH, China), following the manufacturer’s instructions. Whole cell proteins (10 μg) from the *M. bovis* strain or purified recombinant proteins (2 μg) were separated by SDS-PAGE, with a PageRuler pre-stained protein molecular weight marker (#26616, Thermo Fisher Scientific, USA) also included as a reference. The gels were then stained with Coomassie Brilliant Blue Reagent (#C8430, Solarbio, China) or transferred to a polyvinylidene fluoride (PVDF) membrane (#ISEQ00010, Sigma-Aldrich, USA) which was subsequently blocked for 2 hours at room temperature using 5% skim milk (in TBS). After washing the membrane three times (5 minutes each time) with TBST (TBS with 0.05% Tween-20), overnight incubation was performed at 4°C in the presence of a 1:100 dilution of bovine serum (pooled serum of calves experimentally infected with *M. bovis*) or a 1:100 dilution of mouse serum in a primary antibody diluent (#G2025-100ML, Sevicebio, China). This was followed by another washing step with TBST (5 times for 6 minutes each), after which the membrane was incubated at room temperature for 1 hour using a 1:2,000 dilution of horseradish peroxidase (HRP)-conjugated goat anti-bovine antibody (#6030-05, Dako, Denmark) or a 1:10,000 dilution of HRP-conjugated goat anti-mouse antibody (#RGAM001, Proteintech, China). After washing three more times with TBST, for 5 minutes each time, the bound antibodies were detected using Western Bright ECL (#K-12045-D50, Advansta, USA), with images subsequently captured via chemiluminescence using an Amersham imager 600 (Cytiva, USA).

### 2.3 Overlapped stretch PCR and Quantitative Real-Time PCR

A bacterial whole genome DNA extraction kit (#DP302-02, TIANGEN, China) was used for extracting total DNA from strain HB0801. Whole genome sequencing was then performed by amplifying target sequences using a 2× Phanta Max Master Mix (#P515-01, Vazyme, China) as required by the manufacturer along with primers specifically designed for this purpose (**Table 1**). The amplified sequences were subsequently inserted into a *p*ET-30a(+) vector using the ClonExpress II kit (#C112-01, Vazyme, China) to construct recombinant plasmids. MAC-T, BoMac and EBL cells, each at a density of 1×10^6^ cells per well, were seeded into six-well plates and after allowing the cells to adhere overnight, recombinant protein was added to stimulate the cells as required by the experimental protocol. This was followed by total RNA extraction using Trizol reagent (#15596018CN, Thermo Fisher Scientific, USA), after which a corresponding cDNA was synthesized through reverse transcription with a reverse transcriptase kit (#R323-01, Vazyme, China). Finally, qRT-PCR was performed on a qTOWER3 G real-time PCR system (Analytikjena, Germany) using a SYBR Green PCR mixture (#Q711-02, Vazyme, China) and the cDNA as template. **Table 2** provides the list of primers used for amplifying the selected genes.

**Table 1:** Primers used to construct recombinant plasmids.

Primers	Primer sequence
Mbov_0585 F1	ATGGCTGATATCGGATCCATGAAAAATAAATTTAAAAAAA
Mbov_0585 R1	GAGTGCGGCCGCAAGCTTTTAATTAAGTTTAGATATTTTTTGG
Mbov_0585 F2	AGATAGTGAAAAATGGAAAGAATTTATTGCTAATACTTCTGAGTT
Mbov_0585 R2	AACTCAGAAGTATTAGCAATAAATTCTTTCCATTTTTCACTATCT
Mbov_0585 F3	TTATCAATGGTTGGCGTGTCCCGACTTACATATCAGCTAGATTAG
Mbov_0585 R3	CTAATCTAGCTGATATGTAAGTCGGGACACGCCAACCATTGATAA
Mbov_0585 F4	TTTGATAAATGGGTTGATGAAATAAAAGATCTTGACATGATTTCT
Mbov_0585 R4	AGAAATCATGTCAAGATCTTTTATTTCATCAACCCATTTATCAAA
Mbov_0538 F1	CGGATCCATGATAAAAAATAAAAAATATTTTACTTTCATAAT
Mbov_0538 R1	CAAGCTTTTAATTTTTATTATTGCTTAAGTCTACA
Mbov_0538 F2	CCAAAGTAAAAGTTGGGATGTGTTT
Mbov_0538 R2	TTTCAAAAACACATCCCAACTTTTACTTT
Mbov_0538 F3	TACAAGAAAAACTGGTTATGGTTCTTATTTAAC
Mbov_0538 R3	TTATGTTAAATAAGAACCATAACCAGTTTTTCTTGT

**Table 2:** Primers designed for qRT-PCR (16).

Primers	Primer sequence
IL-1β F	GTCATCTTCGAAACGTCCTCC
IL-1β R	TCCTCTCCTTGCACAAAGCTC
GAPDH F	TGGTGAAGGTCGGAGTGAAC
GAPDH R	ATGGCGACGATGTCCACTTT
TNF-α F	CTCCATCAACAGCCCTCTGG
TNF-α R	GAGGGCATTGGCATACGAGT
IL-6 F	ACCCCAGGCAGACTACTTCT
IL-6 R	CCCAGATTGGAAGCATCCGT
IL-8 F	GAAGAGAGCTGAGAAGCAAGATCC
IL-8 R	ACCCACACAGAACATGAGGC

### 2.4 Prediction of membrane lipoprotein based on M.bovis HB0801 genome

The classical and non-classical secretion pathways of membrane lipoproteins were predicted with SignalP 4.1 (http://www.cbs.dtu.dk/services/SignalP/) and SecretomeP 2.0 (http://www.cbs.dtu.dk/services/SecretomeP/), respectively. The conserved domains in proteins were then predicted using the online Conserved Domain Database of the National Center for Biotechnology Information (NCBI) (http://www.ncbi.nlm.nih.gov/Structure/cdd/wrpsb.cgi/). The number of B cell and T cell epitopes was eventually predicted, along with immunogenicity scores, using the IEDB online software (http://www.iedb.org/).

### 2.5 Gene cloning and expression of recombinant proteins

Due to the unique codon usage of mycoplasma species, the UGA codon in the mycoplasma genes was mutated to UGG in *E. coli* to ensure proper expression of the recombinant protein. The overlapping extension PCR technique was then applied to splice gene fragments and obtain the complete sequence for the membrane lipoproteins. This sequence was inserted into the *p*ET-30a(+) plasmid using homologous recombination, with the successful construction of plasmids subsequently confirmed by sequencing. In this study, *p*ET-30a(+) plasmids containing the target sequences for MbovP0585 and MbovP0538 was constructed, while the remaining eight predicted membrane lipoproteins sequences were synthesized by Bioengineering Company (Shanghai) and cloned into the *p*ET-30a(+) vector. Each constructed plasmid was then used for transforming *E.coli* strain BL21(DE3) (#CD601-02, TransGen, China), with the expression of the recombinant protein induced with an appropriate concentration of IPTG(#I8070, Solarbio, China). A nickel column (#17531801, Cytiva, USA) affinity chromatography was eventually used to purify the proteins. The purified recombinant protein was concentrated using an ultrafiltration device(15mL, 10kDa)(#UFC901096, MerckMillipore, Germany). The concentration of purified recombinant protein was determined using a BCA protein assay kit according to the manufacturer’s protocol.

### 2.6 Verification of the recombinant proteins’ immunogenicity

Polyclonal antibodies were obtained from *M. bovis*-infected (positive) and uninfected (negative) cattle serum (22). The immunogenicity of the eight purified recombinant proteins was then assessed by western blotting, as previously described. Additionally, the immunogenicity of recombinant proteins was detected using indirect ELISA. For this purpose, 250 ng of purified protein was used to coat each well of a 96-well plate, and after overnight incubation at 4°C, the wells were washed with PBST. After coating the plates with antigen, each well was blocked with 100 μL of 5% skimmed milk at room temperature for 2 h. The blocking solution was then discarded, and the wells were washed three times with PBST (PBS containing 0.05% Tween-20). Subsequently, 100 μL of *M. bovis*-positive or negative serum (diluted 1:100 in PBS) was added to each well, followed by incubation at 37°C for 1 h. After another PBST wash, 100 μL of HRP-conjugated goat anti-bovine IgG secondary antibody (diluted 1:5000 in PBS) was added, and the plates were incubated at 37°C for 1 h. Following a final PBST wash, 100 μL of TMB substrate solution (#PR1200, Solarbio, China) was added to each well, and the plates were incubated at room temperature for 30 min in the dark. The reaction was stopped by adding 50 μL of stop solution (2 M H_2_SO_4_), and the absorbance was immediately measured at 630 nm (OD_630_) using an EnSpire microplate reader (PerkinElmer, USA).

### 2.7 Preparation of mouse polyclonal antibody against recombinant protein and detection of antibody titer

Four-week-old female BALB/cJGpt mice (Strain no: N000020) were obtained from GemPharmatech (Nanjing, China) and raised in the SPF animal laboratory of Ningxia University. Purified recombinant proteins, for which the concentrations were measured with a Bicinchoninic Acid Assay (BCA) protein quantification kit and adjusted to 100 μg, were then used to immunize the animals. Following the first immunization, equal volumes of Freund ‘s complete adjuvant (#F5881-10ML, Sigma, USA) and purified recombinant protein were mixed and thoroughly emulsified. The mice were subsequently immunized via multi-point subcutaneous injections at the back of the neck. Blood was collected prior to immunization to serve as the negative control. The second immunization was administered two weeks after the first, and was followed by a third immunization one week later. For the second and third immunizations, the protein solution was emulsified with Freund’s incomplete adjuvant(#F5506-10ML, Sigma, USA), with the injection then performed as described above. One week after the third immunization, serum was collected for storage at -20°C.

The purified recombinant protein was diluted in coating buffer (Na_2_CO_3_/NaHCO_3_/ddH_2_O) and used to coat each well of ELISA plates at 100 ng/well. After overnight incubation at 4°C, the plates were blocked with 5% skimmed milk and washed with PBST. Mouse polyclonal antibodies against each protein were serially diluted in PBS (ranging from 1:100 to 1:1,638,400) and added to individual wells. Pre-immune mouse serum was included as the negative control group. Following 1 h incubation at 37°C, the plates were washed with PBST and incubated with HRP-conjugated goat anti-mouse IgG (Proteintech; 1:20,000 dilution) for 1 h at 37°C. After final washing, 100 μL of TMB substrate solution was added to each well and allowed to develop at room temperature for 30 min in the dark. The reaction was terminated by adding 50 μL of stop solution, and the absorbance was measured at 630 nm (OD₆₃₀) using an EnSpire microplate reader.

### 2.8 Subcellular localization of M. bovis’s membrane lipoprotein

Strain HB0801 was inoculated in PPLO medium for 48 h at 37 °C, after which its membrane proteins were separated from the cytoplasmic proteins using a mycoplasma membrane protein extraction kit (#BB-31515, Bestbio, China). Separately, 10 mL of an *M. bovis* culture in the logarithmic phase was centrifuged for 10 min and at 14,000 g, with the resulting pellet subsequently resuspended in PBS buffer to yield whole cell proteins. These different proteins (i.e., membrane proteins, cytoplasmic proteins and whole cell proteins) were then mixed with 6×protein loading buffer prior to analysis by western blotting as described above.

### 2.9 Verifying the secretion of the selected membrane lipoprotein

Whole-cell protein extracts of *M. bovis* were prepared as described in Section 2.2. The supernatant was then filtered through a 0.1-μm filter to remove cell debris, followed by concentration to 1 mL with an ultrafiltration device(15mL, 3kDa)(#VS15T91, Sartorius, USA) A 100 μL aliquot of the concentrated supernatant was mixed with 6×protein loading buffer and denatured at 100°C for 10 min in a heating block. Western blotting was eventually performed to detect the secretion of eight membrane lipoproteins.

To investigate the secretion profile of MbovP0592, culture supernatants of *M. bovis* strain HB0801 were collected at 12, 24, 36, and 48 h post-inoculation (10 mL per time point). The samples were centrifuged at 14,000 × g for 10 min at room temperature to remove bacterial cells, followed by filtration through 0.1 μm membranes for further clarification. The clarified supernatants were then concentrated to 500 μL using ultrafiltration device(15mL, 3kDa). The secretion kinetics of MbovP0592 protein were subsequently analyzed by western blotting as described above. The known secreted membrane lipoprotein MbovP0475 and concentrated PPLO medium (equal volume) served as positive and negative controls, respectively.

### 2.10 Removal and detection of endotoxins and purity analysis in recombinant proteins

To 1 mL of protein sample, 100 μL of a protein liquid phase deendotoxin kit (#GMS 30046.2.1, GENMED, USA) was added to remove the endotoxin. The mixture was shaken for 30 minutes at 4 °C, and after being kept for 10 minutes in a water bath at 37 °C, a 10-minute centrifugation was performed at 16,000 g. This yielded an upper liquid phase containing the membrane proteins and a lower liquid phase consisting of the cytoplasmic proteins. The removal efficiency of endotoxin was eventually assessed from a standard curve and according to the instructions of Limulus test Kit (#EC64405, BIOENDO, China). For this experiment, the procedure was repeated three times for each sample. Protein purity was quantified using Image Lab™ (v6.1, Bio-Rad) software. Band intensities from SDS-PAGE gels were analyzed by densitometry, with target protein purity calculated as the percentage of the target band intensity relative to the total lane intensity after background subtraction.

### 2.11 Deendotoxin recombinant protein stimulates cells

Cells were seeded in 6-well plates at a density of 1×10⁶ cells per well and allowed to adhere. The culture medium was aspirated, and the cells were gently washed twice with phosphate-buffered saline (PBS).Recombinant protein (endotoxin-free) was diluted in fresh cell culture medium to final concentrations of 3 μg/mL, 5 μg/mL, and 10 μg/mL, followed by sterilization through a 0.22 μm filter. Subsequently, 2 mL of the recombinant protein-containing medium was added to each well of the 6-well plate for further experimental treatments.

### 2.12 Statistical analyses

Statistical analyses were performed in GraphPad Prism version 8 (GraphPad Software, La Jolla, CA, USA). Image Lab™ was used for analyses of purity of protein bands. The data were expressed as mean ± standard error of the mean (SEM). Data are reported as means ± SD, with *p* values < 0.05 being considered significant,and *p* values <0.05, 0.01, 0.001 were marked respectively as *, **, and *** in the figures. All data were obtained from three independent biological replicates.

## 3 Results

### 3.1 Analysis of membrane lipoprotein based on M. bovis HB0801 genome

In studying *M. bovis*’s genome, the current team previously identified 39 membrane lipoproteins. There are 11 membrane lipoproteins have been functionally characterized and published (14). Among the remaining 28 membrane lipoproteins (**Supplementary Table 1**), we performed bioinformatics analysis on 10 selected candidates to determine their SignalP-TM scores, SceP scores, B cell and T cell epitopes as well as immunogenicity scores (**Table 3**). Of these, MbovP0274 exhibited the highest number of B cell epitopes, while MbovP0289 contained the most T cell epitopes. Additionally, five membrane lipoproteins were found to contain conserved domains. For instance, MbovP0538 contained the TIGRFAMs-TIGR04313 domain, an aromatic cluster surface protein domain that may be involved in enzymatic catalysis or serve as a cell surface receptor by binding to specific substrates. Similarly, MbovP0119 consisted of cysteine proteinases domain, suggesting its potential role in protein degradation, cell cycle regulation and immune response. Finally, MbovP0289 possesses an CtpA and belongs to the S41 family peptidase, which may be involved in protein degradation both inside and outside the cell, thus supporting normal cell metabolism and cell cycle regulation.

**Table 3:** Details on the 10 predicted membrane lipoproteins as determined by bioinformatics analyses.

ORF	SignalP-TM Score	SecP Score	B Cell Epitope	T Cell Epitope	Immunogenicity score	Domain
Mbov_0393	0.465	0.895	15	13	0.450	No
Mbov_0274	0.53	0.825	26	9	0.201	No
Mbov_0538	0.506	0.861	8	8	0.242	TIGRFAMs-TIGR04313
Mbov_0119	0.537	0.839	17	18	1.121	Cysteine proteinases
Mbov_0289	0.576	0.888	19	24	0.547	CtpA
Mbov_0585	0.583	0.825	13	12	-0.202	No
Mbov_0659	0.609	0.819	19	22	0.414	TAIL-SPECIFIC PROTEINASES
Mbov_0084	0.622	0.839	17	8	1.128	No
Mbov_0536	0.635	0.646	10	3	0.924	Aromatic Binding Domain
Mbov_0592	0.583	0.834	13	6	-0.210	No

### 3.2 Recombinant protein expression and immunogenicity verification

**Figure 1:**
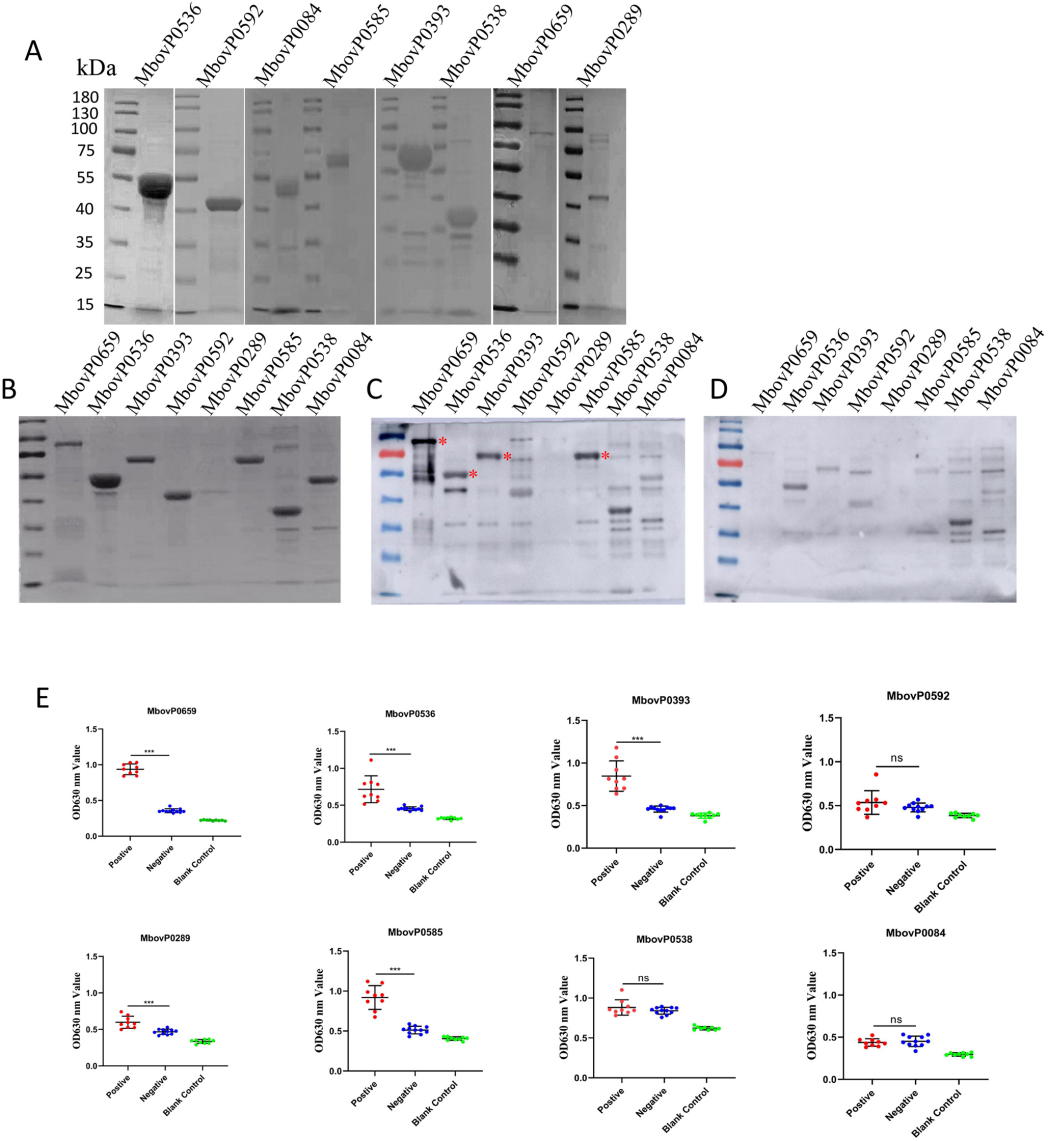
Purification of membrane lipoproteins and immunogenicity test. **(A)** Purification of eight predicted membrane lipoproteins by nickel affinity chromatography. **(B)** The predicted membrane lipoproteins were separated by SDS-PAGE prior to staining with Coomassie brilliant blue. **(C-D)** *M. bovis*-positive serum **(C)** or negative serum **(D)** from cattle were incubated before using horseradish peroxidase (HRP)-conjugated goat anti-bovine antibody as secondary antibody to detect the immunogenicity of membrane lipoproteins by Western blotting. (* Labeled with immunogenic response bands) **(E)** Indirect ELISA was performed to assess the immunogenicity of membrane lipoproteins. *P < 0.05, **P < 0.01, ***P < 0.001; ns, no significance.

SDS-PAGE analysis confirmed the correct expression of the recombinant protein at the expected molecular weights (**Figure 1A**). However, the expression levels of MbovP0274 and MbovP0119 in the supernatant after cell lysis were too low for large-scale preparation. The purified recombinant proteins were then resolved via SDS-PAGE (**Figure 1B**) and subsequently tested with bovine serum. In this case, western blotting analysis showed that MbovP0659, MbovP0536, MbovP0393 and MbovP0585 reacted with serum from *M. bovis*-infected cattle (**Figure 1C**), while bovine negative serum did not show any reaction with the recombinant proteins (**Figure 1D**). These results, which were subsequently confirmed by ELISA (**Figure 1E**), indicated that cattle infected with *M. bovis* could produce specific antibodies against the above four recombinant proteins, hence identifying them as the immunogenic components of *M. bovis*. Analysis of the experimental results indicated that MbovP0289 displayed no detectable immunogenicity via Western blotting, whereas ELISA confirmed its immunogenic potential. We propose that the denaturation of the antigen during electrophoresis in Western blotting may have disrupted its native conformation, limiting antibody recognition to linear epitopes. If the target antibodies were specific to conformation-dependent epitopes (e.g., three-dimensional structural motifs), they would fail to bind the denatured antigen. In contrast, the ELISA employed native recombinant protein for coating, preserving conformational epitopes and enabling proper antibody-antigen interaction. Furthermore, ELISA demonstrates superior sensitivity compared to Western blotting, particularly for low-abundance antibodies, such as the polyclonal antibodies derived from clinical bovine serum used in this study.

### 3.3 Determination of anti-recombinant proteins polyclonal antibody titers in mouse serum

**Figure 2:**
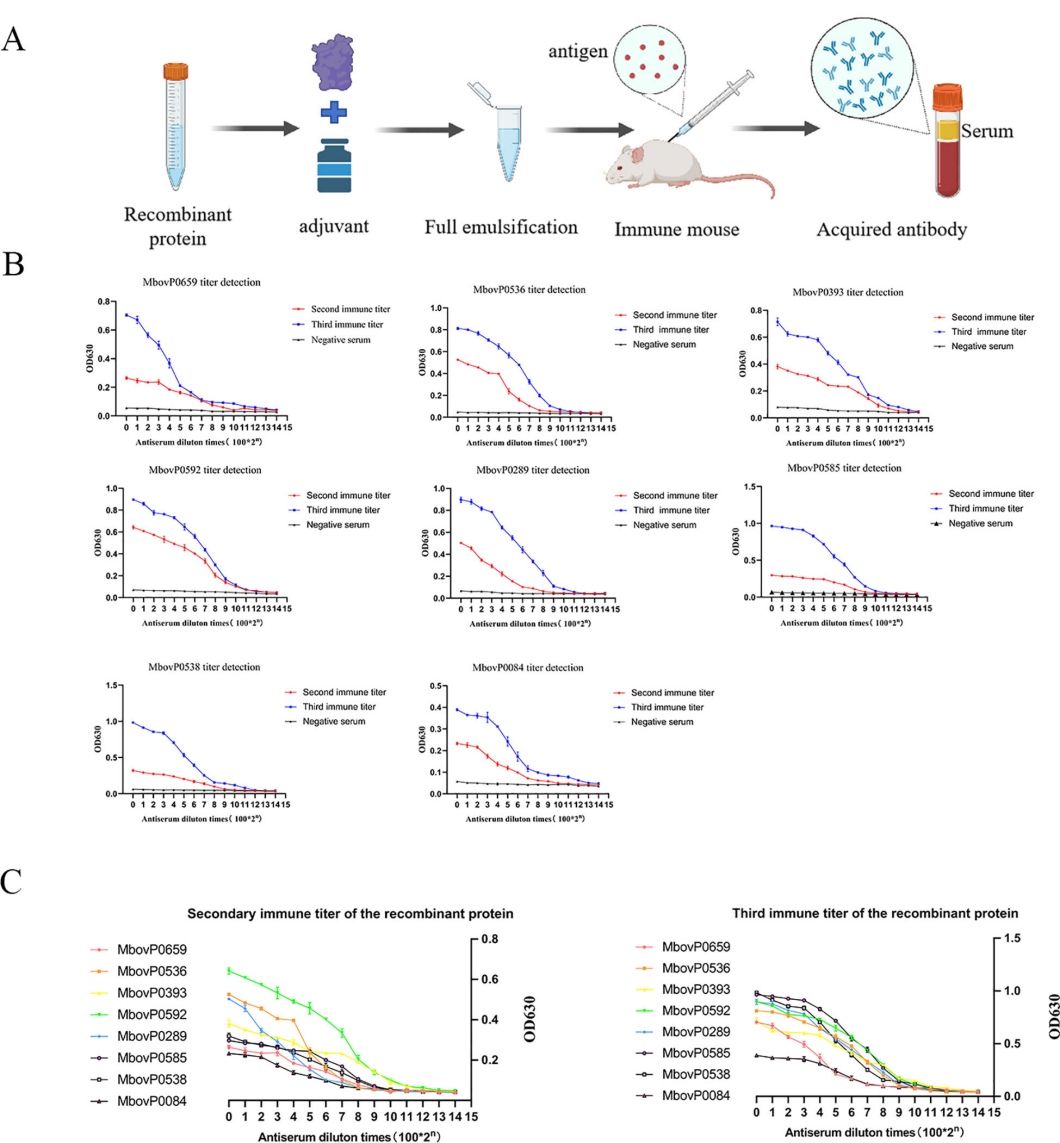
Mouse anti-recombinant proteins polyclonal antibody preparation flow chart and antibody titer detection. **(A)** Flow chart for the preparation of mouse polyclonal antibodies **(B)** The titer of secondary and tertiary immune antibodies was determined using indirect ELISA. The mouse polyclonal antibodies against eight proteins were diluted with PBS from 1:100 to 1:1,638,400 times for detection. Red represents secondary immunization; blue represents tertiary immunization and black represents negative control. **(C)** Comparison chart of antibody titers of 8 recombinant proteins.

To facilitate downstream applications of the recombinant proteins, we generated mouse polyclonal antisera against eight recombinant proteins following the immunization protocol outlined in (**Figure 2A**). Serum antibody titers were evaluated by indirect ELISA after the second and third immunizations. As shown in (**Figure 2B**), all eight antisera exhibited endpoint titers >2^10^ following the third immunization, meeting the predefined threshold for successful antibody production. Notably, the third immunization consistently elicited higher antibody titers compared to the second immunization, demonstrating enhanced immunogenicity and confirming the necessity of tertiary immunization for optimal polyclonal antibody generation. Furthermore, we analyzed and compared the antibody titers following the second and third immunizations of the eight recombinant proteins(**Figure 2C**), with the results provided (**Supplementary Table 2**).

### 3.4 Secretory verification and subcellular localization of M. bovis membrane lipoprotein

**Figure 3:**
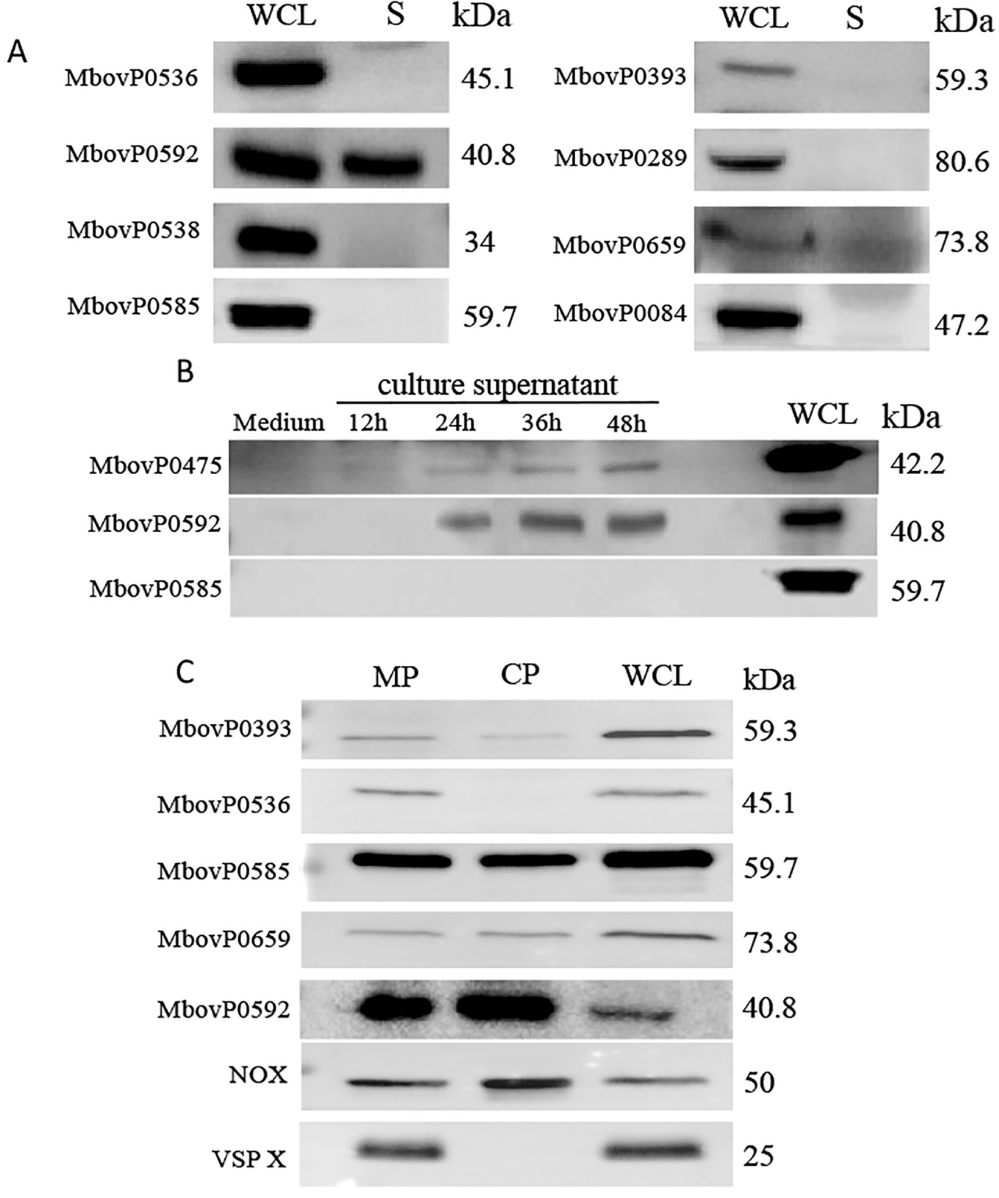
Secretory verification and subcellular localization. **(A)** Supernatants (S) and whole cell lysates (WCL) from *M. bovis* culture were separated by SDS-PAGE before being transferred to a PVDF membrane. Proteins in the supernatant were detected using polyclonal antibodies against rMbovP0536, rMbovP0592, rMbovP538, rMbovP0585, rMbovP0393, rMbovP0289, rMbovP0659 and rMbovP0084. **(B)** Visualization of MbvoP0592 secreted in the culture supernatant. The supernatant from a 12-hours, 24-hours, 36-hours and 48-hours culture of *M. bovis* were concentrated before using the antiserum against rMbovP0592 to detect the protein in the supernatant. In this case, the secretory membrane lipoprotein rMbovP0475 was used as a positive control, while the non-secretory membrane lipoprotein rMbovP0585 was used as a negative control. **(C)** Western blotting was performed for detecting MbovP0393, MbovP0536, MbovP0585, MbovP0592 and MbovP0659 expression in the cytoplasm and cell membrane of *M. bovis*. MP represents cell membrane protein, CP represents cytoplasmic protein, and WCL represents whole cell protein. VSP X protein and NOX protein used as controls.

Determination of membrane lipoprotein secretion in *M. bovis*, their culture supernatant presence was first examined. Interestingly, only MbovP0592 was detected in the culture supernatant (**Figure 3A**), and to observe its secretion dynamics *in vitro*, its expression in *M. bovis*’s culture supernatant was tracked over time (**Figure 3B**) using MbovP0475 (a secreted lipoprotein) as a positive control and MbovP0585 (a membrane protein) as a negative control (15). Western blotting indicated that MbovP0592 was detectable in the supernatant after 24 hours of culture, with its expression also increasing gradually over time. Through the aforementioned experimental validation of the immunogenicity and secretory properties of membrane lipoproteins, we have identified one secretory membrane lipoprotein (MbovP0592) and four immunogenic membrane lipoproteins (MbovP0393, MbovP0536, MbovP0585, MbovP0659). Given their favorable biological characteristics and high research value, these five proteins will be further characterized in subsequent studies for scientific investigation purposes. Following separation of the cell membrane and cytoplasmic fractions of *M. bovis*, one secretory lipoprotein and four immunogenic proteins were subcellular localized using Western blotting analysis. (**Figure 3C**). In this case, it was found that MbovP0393, MbovP0536, MbovP0585, MbovP0659 and MbovP0592 were all contained on *M. bovis*’s membrane. Furthermore, beside MbovP0536, the four others membrane lipoproteins were also expressed in the cytoplasm. As expected, NOX (23), a membrane-associated protein, was observed in both the cytoplasmic and membrane fractions, while VSP X, a membrane-specific protein, was only found in the membrane fraction (24), which were used as controls. Additionally, entire images for the secretory verification(**Supplementary Figures S1A, S1B**) and subcellular localization(**Supplementary Figure S1C**) of *M. bovis* membrane lipoproteins will be presented in supplementary figures, enabling analysis of nonspecific reactions.

### 3.5 Endotoxin removal from recombinant proteins

**Figure 4:**
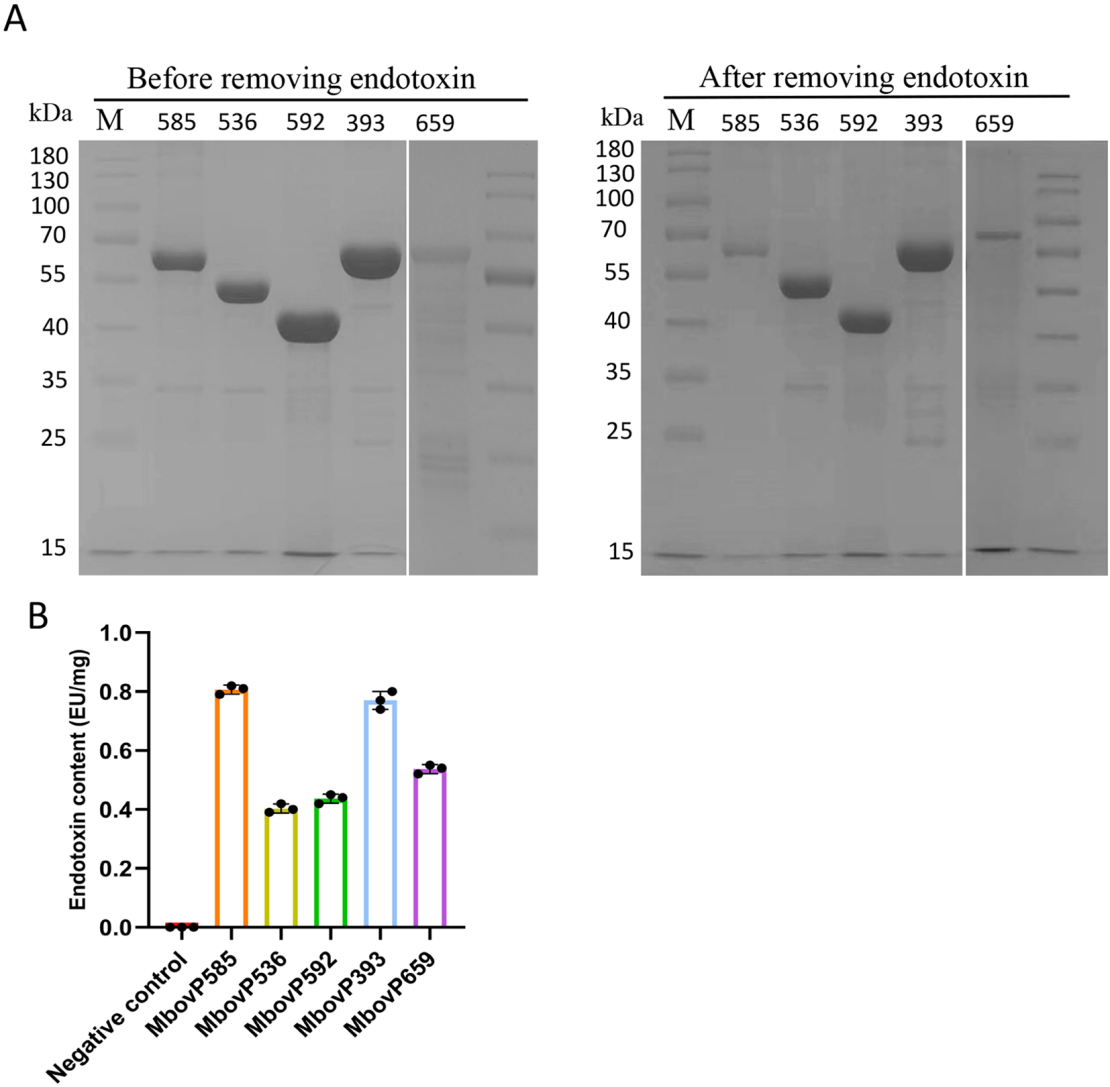
Removal of endotoxin from recombinant proteins. **(A)** SDS-PAGE was used to detect the protein purity of recombinant proteins after endotoxin removal. **(B)** Limulus reagent was used to detect the endotoxin content of recombinant proteins after endotoxin removal. Each protein was subjected to three repeated experiments.

Endotoxin removal was performed on the five recombinant proteins expressed in *E. coli* using an endotoxin removal kit. This resulted in a high protein purity as reflected in SDS-PAGE analysis (**Figure 4A**), with Image Lab™ software confirming purity levels above 90%. Endotoxin levels were then assessed with a Limulus reagent endotoxin test kit which showed that the endotoxin concentrations in the recombinant proteins were reduced to below 1 EU/mg, thus meeting the requirements for subsequent cell experiments (**Figure 4B**).

### 3.6 Recombinant proteins stimulated the expression of pro-inflammatory cytokines in epithelial cells

**Figure 5:**
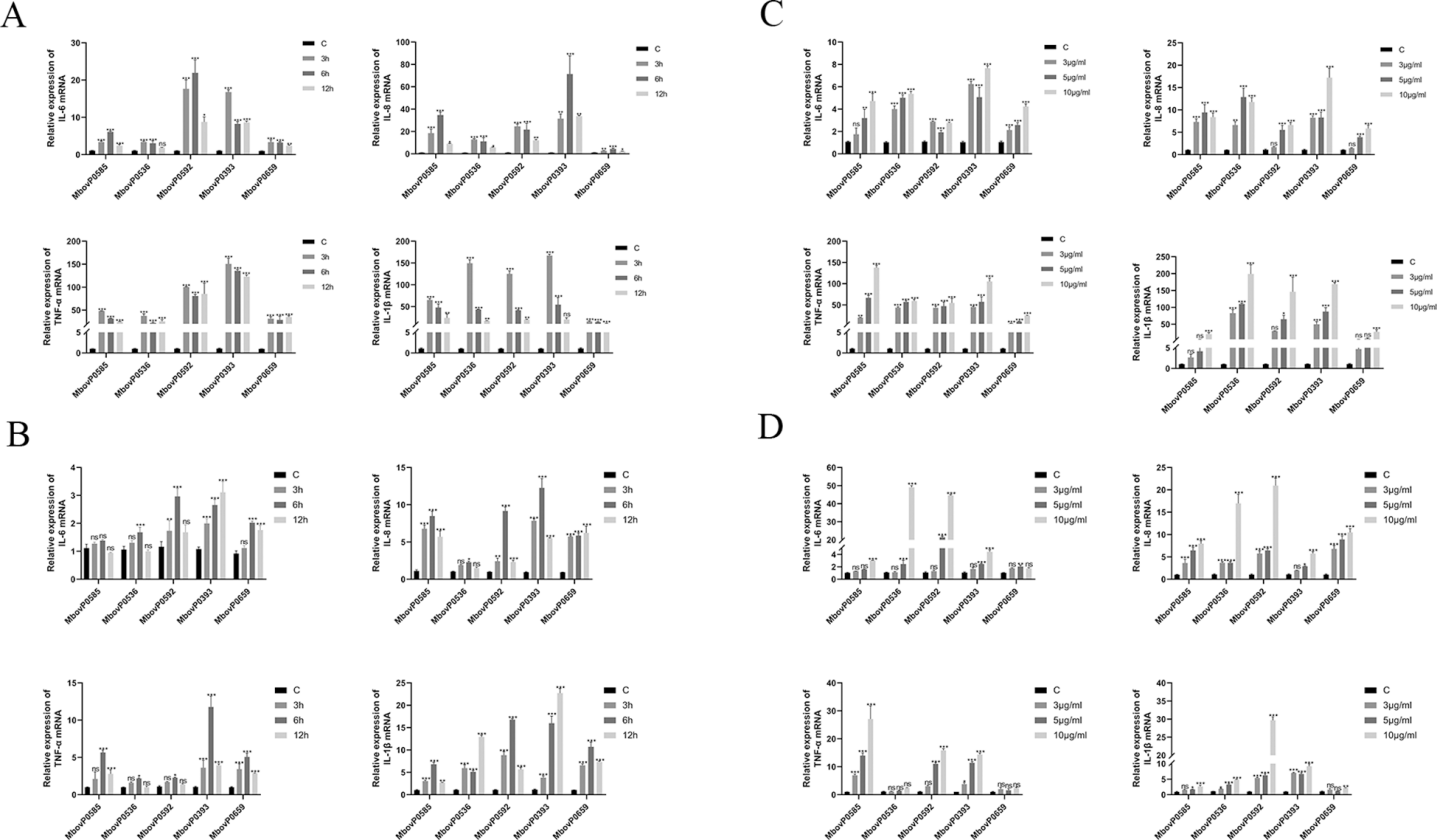
Recombinant proteins induced the expression of pro-inflammatory cytokines in epithelial cells. The concentration of recombinantproteins was 5 μg/mL, and the expression level of pro-inflammatory cytokines in **(A)** MAC-T cells and **(B)** EBL cells were assessed after stimulation for 3, 6 and 12 hours. Different concentrations (3, 5 and 10 μg/mL) of recombinant proteins were used to treat **(C)** MAC-T cells for 3 hours and **(D)** EBL cells for 6 hours before assessing the expression level of pro-inflammatory cytokines. *P < 0.05, **P < 0.01, ***P < 0.001; ns, no significance.

Endotoxin-free recombinant proteins were used at a concentration of 5 μg/mL to stimulate two types of bovine epithelial cells, namely MAC-T and EBL, for 3, 6 and 12 hours. Total RNA was then extracted from the cells to determine the expression level of IL-1β, TNF-α, IL-8 and IL-6 in MAC-T (**Figure 5A**) and EBL (**Figure 5B**) using qRT-PCR. In comparison with the control, the four pro-inflammatory cytokines were expressed at significantly higher levels after recombinantproteins stimulation across various time points, especially in the case of MAC-T cell lines. In particular, the relative mRNA expression levels of these cytokines generally peaked at 3 hours post-treatment in MAC-T cells, whereas in EBL cells, higher mRNA expression levels were observed at 6 hours after treatment. Therefore, 3 hours and 6 hours were selected as the optimal stimulation times for MAC-T and EBL cells, respectively. The two cell lines were then stimulated with 3, 5 or 10 μg/mL of endotoxin-free recombinant proteins for 3 hours (**Figure 5C**) or 6 hours (**Figure 5D**) before determining the relative expression of pro-inflammatory cytokines using qRT-PCR. Compared with the control group, there was an increase in the mRNA expression of the pro-inflammatory with all concentrations of recombinant proteins, with higher expression observed at a concentration of 10 μg/mL in both cell lines.

### 3.7 Recombinant proteins stimulated the expression of proinflammatory cytokines in macrophages

**Figure 6:**
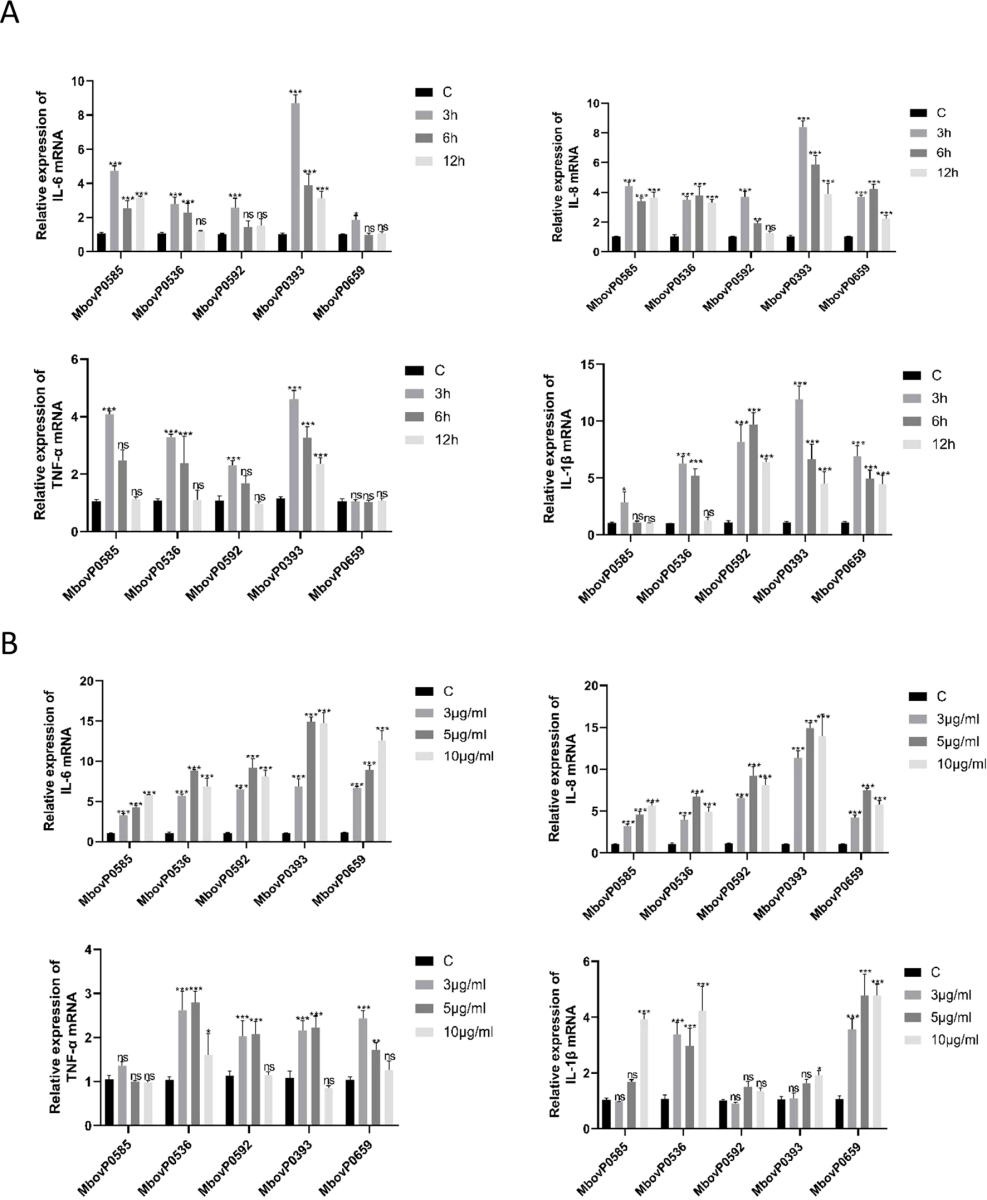
Recombinant proteins induced the expression of pro-inflammatory cytokines in macrophages. **(A)** Recombinant proteins (5 μg/mL) were used to treat BoMac cells for different durations (3, 5 and 10 hours) before detecting the level of pro-inflammatory cytokines. **(B)** Different concentrations (3, 5 and 10 μg/mL) of recombinant proteins were used to treat BoMac cells for 3 hours before assessing the level of pro-inflammatory cytokines. *P < 0.05, **P < 0.01, ***P < 0.001; ns, no significance.

To further investigate the inflammatory response induced by recombinant proteins in host macrophages, BoMac cells were selected for further experiments. Macrophages play a central role in innate immunity, and hence, are highly indicative of inflammatory responses. Building on the experimental approach from Section 3.5, we further investigated the optimal time and concentration conditions for five endotoxin-free recombinant proteins to stimulate inflammatory cytokine expression in BoMac cells. Following total RNA extraction, we quantified IL-1β, TNF-α, IL-8, and IL-6 expression levels in BoMac cells using qRT-PCR. Results indicated that the optimal treatment conditions were 3 hours at 10 μg/mL for MbovP0585, MbovP0536, and MbovP0393, while MbovP0592 and MbovP0659 showed optimal response at 3 hours with 5 μg/mL concentration (**Figure 6A, B**).

## 4 Discussion

*M. bovis* was first discovered in the United States in 1961 (25), and today, it is not only prevalent worldwide, but it is also recognized as a major pathogen in cattle, contributing to nearly 10% of deaths in the cattle breeding industry (26). With the complete sequencing of the *M. bovis* genome, it was found that strain HB0801 encodes 808 genes, of which 762 code for proteins (21). Among these, membrane-associated proteins, especially lipoproteins, are recognized as factors involved in stimulating the immune response (4). In this context, a previous study demonstrated that a subunit vaccine targeting *M. bovis* was linked with various immunogenic proteins, including DeoB, O256, PepQ, LppA, OppA, P81, P48, Tuf, PepA, PdhA and GAPDH (27). However, despite a strong humoral immune response marked by IgG1 and IgG2 serum responses (28), this subunit vaccine did not protect against *M. bovis* challenges. Thus, identifying *M. bovis* membrane lipoproteins capable of inducing cytokine expression in host epithelial cells and macrophage may provide new ideas for vaccine development.

Due to the lack of a cell wall, the secretome of *M. bovis* consists of proteins released freely into the extracellular environment (extracellular proteome), surface-exposed proteins (surface proteome) and proteins embedded within extracellular vesicles (vesicle-associated proteome) (29). Both surface and extracellular proteins are believed to be involved in host-mycoplasma interactions. Indeed, previous studies indicated that secretory membrane lipoproteins are crucial in *M. bovis*’s virulence, regulating the host cells’ vitality and influencing bacterial growth and metabolism. However, there are several challenges in studying the secretory membrane lipoproteins of *M. bovis* (1): the lack of effective genetic manipulation tools as existing gene knockouts rely on transposon-based random insertion which does not allow for targeted knockout (2); the genome contains many proteins with unknown functions, thus necessitating high-throughput methods for screening related biological phenotypes rather than direct analysis (3); the culture medium required for the growth of *M. bovis* was rich in serum and yeast extracts, thus making it difficult to exclude exogenous protein contamination from the supernatant; and (4) the absence of animal models as current research is predominantly limited to the cellular level, and hence, *in vitro* studies may not fully reflect the actual case. In this study, among the 10 predicted membrane lipoproteins, only MbovP0592 was found to be highly expressed in the culture supernatant of *M. bovis*. It is currently believed that mycoplasma species secreted lipoproteins either through cleavage of their signal peptide or by embedding them in extracellular vesicles (30, 31). Furthermore, MbovP0592 was found in the membrane fraction of *M. bovis*, hence suggesting it may be released from the membrane to the extracellular environment. However, further experiments would be required to confirm this hypothesis. In our study, the recombinant MbovP0592 protein exhibited secretory properties and pro-inflammatory activity *in vitro*. However, no immunogenicity was detected in sera from *M. bovis* -infected cattle. Unlike the recombinant protein, native virulence factors in *M. bovis* are highly complex and variable. We hypothesize that MbovP0592 may undergo post-translational modifications (e.g., glycosylation, lipidation, or phosphorylation) that mask its immunogenic epitopes, thereby limiting antigenic exposure. Alternatively, phase variation in the MbovP0592 gene could lead to dynamic epitope alterations during infection, preventing the host from generating stable antibody responses. Additionally, MbovP0592 might directly suppress B-cell activation or interfere with antigen presentation pathways (e.g., by inhibiting MHC-II signaling), thereby blocking the production of specific antibodies. Furthermore, discrepancies between experimental infection models and natural pathogenic conditions—including variations in serum collection methods and timing—may affect antibody levels and contribute to the observed experimental differences. Future studies should investigate the regulatory effects of MbovP0592 on B-cell and T-cell activation, as well as antigen-presenting cell function, to elucidate its immune evasion mechanisms.

In *Mycoplasma*, the structure and function of signal peptides and lipoboxes exhibit a certain degree of conservation and specificity compared to those in conventional bacteria. mycoplasma lipoprotein signal peptides typically comprise an N-terminal positively charged region (n-region), a hydrophobic core (h-region), and a C-terminal lipobox, resembling the Sec secretion pathway signal peptides of canonical bacteria (e.g., Escherichia coli) (32). However, mycoplasma signal peptides are generally shorter than those of bacteria with cell walls, a feature likely attributed to their minimal genome. In *Mycoplasma* species, the basic structure of the lipobox is highly conserved. For example, all non-homologous lipoproteins in *Mycoplasma pneumoniae (M. pneumoniae*) share a core lipobox sequence of [LVI][ASTVI][GAS]C (33). Compared to conventional bacteria, mycoplasma lipoboxes retain only the critical cysteine (C) residue at the core position, while exhibiting greater flexibility at other sites (34). Most current lipoprotein prediction tools are trained on data from conventional bacteria with cell walls and may exhibit reduced sensitivity to the shorter signal peptides or atypical lipoboxes in *Mycoplasma*. However, due to limitations in genetic tools and experimental protocols for *Mycoplasma* research, traditional software remains widely applied for lipoprotein prediction in these organisms. For instance: Berry et al. utilized SignalP to predict signal peptides in surface proteins of *M. pneumoniae* (35).Raheem et al. employed SignalP 5.0 to identify a signal peptide in the *M. bovis* protein MbovP467 between residues 27 and 28 amino acids (36).Zhao et al. applied SignalP 4.1 to screen classical secretory proteins in the *M. bovis* HB0801 strain and experimentally validated two secretory lipoproteins, MbovP0280 and MbovP0475 (14). These examples underscore that experimental validation of prediction results is particularly critical for mycoplasma lipoprotein studies.

The inflammatory response is crucial to the pathogenesis of *M. bovis* disease. In this context, Bassel ‘s study found that inflammatory stimulation in calves with *M. bovis* respiratory disease led to more severe symptoms of *M. bovis* pneumonia (37). Additionally, reducing the pulmonary inflammatory response in calves after *M. bovis* infection has been shown to decrease the likelihood of necrotizing bronchopneumonia (38). Both *in vitro* and *in vivo* studies also confirmed that *M. bovis* induces the production of pro-inflammatory chemokines and cytokines in host cells, resulting in immune-mediated pathology (39, 40). However, at the same time, *M. bovis* can evade immune clearance by the host’s immune system by promoting the secretion of anti-inflammatory cytokines (1). Therefore, the inflammatory response is integral to the pathogenesis of *M. bovis*. In this study, the expression of pro-inflammatory cytokines (IL-1β, TNF-α, IL-8 and IL-6) in host epithelial cells and macrophages as a result of stimulation by five immunogenic membrane lipoproteins were explored. Given that *M. bovis* causes pneumoniae and mastitis in beef and dairy cattle, BoMac, EBL and MAC-T cells were selected to perform further experiments. MAC-T cells, a component of mammary gland immune function, are particularly important during the early infection stage, especially since they can present immunogenic pathogens after infection (41, 42). This study used qRT-PCR to assess the expression of pro-inflammatory cytokines in MAC-T cells following stimulation by recombinant proteins. The results indicated that cytokines expression was highest at 10 μg/mL protein and 3 hours post-treatment, and hence, were consistent with previous findings showing elevated cytokines expression in MAC-T cells infected with *M. bovis* for 4 hours (19). These results also suggest that MAC-T cells can initiate an inflammatory response early during pathogen infection. Additionally, it was found that recombinant proteins stimulated the release of pro-inflammatory cytokines in EBL cells, with the cytokine’s expression peaking at 6 hours post-treatment. Macrophages, known to be crucial for clearing bacteria, releasing cytokines and antigen presentation, also responded with high cytokines expression after stimulating BoMac cells for 3 hours with recombinant proteins. However, the cytokines expression profile in BoMac cells differed significantly from that of MAC-T cells, possibly due to differences between cell types (43). Although this study validated the function of predicted membrane lipoproteins and characterized their ability to stimulate host cell inflammatory responses, it was limited by the lack of research on the mechanisms underlying this response. In addition, pro-inflammatory responses induced by lipoproteins at the bacterial level were not verified.

## Data Availability

The raw data supporting the conclusions of this article will be made available by the authors, without undue reservation.
