# Supplementary material for: Novel Mycoplasma bovis membrane lipoproteins induce the inflammatory response of host epithelial cells and macrophage
Source: Front Immunol. 2025 Jun 9;16:1580436. doi: 10.3389/fimmu.2025.1580436 (PMC12183183; doi:10.3389/fimmu.2025.1580436)
Supplement: Supplementary file 1 [file Table1.docx]

**Table S1** The predicted membrane lipoproteins of *Mycoplasma bovis*

| **Number** | **ORF** | **MW（kDa）** | **SecP Score** | **B Cell Epitope** | **T Cell Epitope** | **SignalP-TM Score** | **Domain** |
| --- | --- | --- | --- | --- | --- | --- | --- |
| 1 | Mbov_0393 | 59.3 | 0.895 | 15 | 13 | 0.465 | No |
| 2 | Mbov_0274 | 66.5 | 0.825 | 26 | 9 | 0.53 | No |
| 3 | Mbov_0538 | 34 | 0.861 | 8 | 8 | 0.506 | TIGRFAMs-TIGR04313 |
| 4 | Mbov_0119 | 75.4 | 0.839 | 17 | 18 | 0.537 | Cysteine proteinases |
| 5 | Mbov_0289 | 80.6 | 0.888 | 19 | 24 | 0.576 | CtpA |
| 6 | Mbov_0585 | 59.7 | 0.825 | 13 | 12 | 0.583 | No |
| 7 | Mbov_0659 | 73.8 | 0.819 | 19 | 22 | 0.609 | TAIL-SPECIFIC PROTEINASES |
| 8 | Mbov_0084 | 47.2 | 0.839 | 17 | 8 | 0.622 | No |
| 9 | Mbov_0536 | 45.1 | 0.646 | 10 | 3 | 0.635 | Aromatic Binding Domain |
| 10 | Mbov_0592 | 40.3 | 0.834 | 13 | 6 | 0.583 | No |
| 11 | Mbov_0477 | 72.5 | 0.826 | 25 | 9 | 0.483 | No |
| 12 | Mbov_0374 | 83.3 | 0.896 | 22 | 15 | 0.500 | No |
| 13 | Mbov_0570 | 85.5 | 0.837 | 24 | 16 | 0.532 | No |
| 14 | Mbov_0016 | 51.2 | 0.813 | 14 | 17 | 0.559 | ABC transporter protein |
| 15 | Mbov_0546 | 86.5 | 0.837 | 23 | 10 | 0.560 | No |
| 16 | Mbov_0350 | 82.8 | 0.921 | 28 | 17 | 0.566 | No |
| 17 | Mbov_0217 | 53.5 | 0.855 | 11 | 9 | 0.587 | No |
| 18 | Mbov_0654 | 28.4 | 0.937 | 3 | 2 | 0.618 | No |
| 19 | Mbov_0347 | 30.2 | 0.894 | 1 | 2 | 0.623 | No |
| 20 | Mbov_0447 | 30 | 0.932 | 4 | 3 | 0.652 | No |
| 21 | Mbov_0292 | 50.1 | 0.766 | 10 | 0 | 0.665 | No |
| 22 | Mbov_0469 | 25.2 | 0.957 | 4 | 1 | 0.666 | No |
| 23 | Mbov_0525 | 63.5 | 0.981 | 1 | 0 | 0.693 | No |
| 24 | Mbov_0461 | 21.5 | 0.873 | 5 | 4 | 0.708 | No |
| 25 | Mbov_0473 | 23.7 | 0.925 | 4 | 2 | 0.709 | No |
| 26 | Mbov_0465 | 85.7 | 0.869 | 25 | 15 | 0.55 | Transglutaminase-like superfamily |
| 27 | Mbov_0339 | 34.8 | 0.932 | 4 | 4 | 0.613 | No |
| 28 | Mbov_0292 | 50.1 | 0.766 | 10 | 9 | 0.665 | No |
