# Supplementary material for: Novel Mycoplasma bovis membrane lipoproteins induce the inflammatory response of host epithelial cells and macrophage
Source: Front Immunol. 2025 Jun 9;16:1580436. doi: 10.3389/fimmu.2025.1580436 (PMC12183183; doi:10.3389/fimmu.2025.1580436)
Supplement: Supplementary file 2 [file Table2.docx]

**Table S2** Result of Antibody Titers Following Secondary and Tertiary Immunizations in Murine Polyclonal Antibody Production

| **Antigen** | **Second immune titer** | **Third immune titer** |
| --- | --- | --- |
| MbovP0659 | 1:25,600 | 1:4,096,000 |
| MbovP0536 | 1:12,800 | 1:102,400 |
| MbovP0393 | 1:102,400 | 1:4,096,000 |
| MbovP0592 | 1:102,400 | 1:102,400 |
| MbovP0289 | 1:12,800 | 1:102,400 |
| MbovP0585 | 1:25,600 | 1:102,400 |
| MbovP0538 | 1:12,800 | 1:204,800 |
| MbovP0084 | 1:6,400 | 1:102,400 |
