# Supplementary material for: Novel Mycoplasma bovis membrane lipoproteins induce the inflammatory response of host epithelial cells and macrophage
Source: Front Immunol. 2025 Jun 9;16:1580436. doi: 10.3389/fimmu.2025.1580436 (PMC12183183; doi:10.3389/fimmu.2025.1580436)
Supplement: Supplementary file 3 [file Table3.docx]

Supplementary Material

**
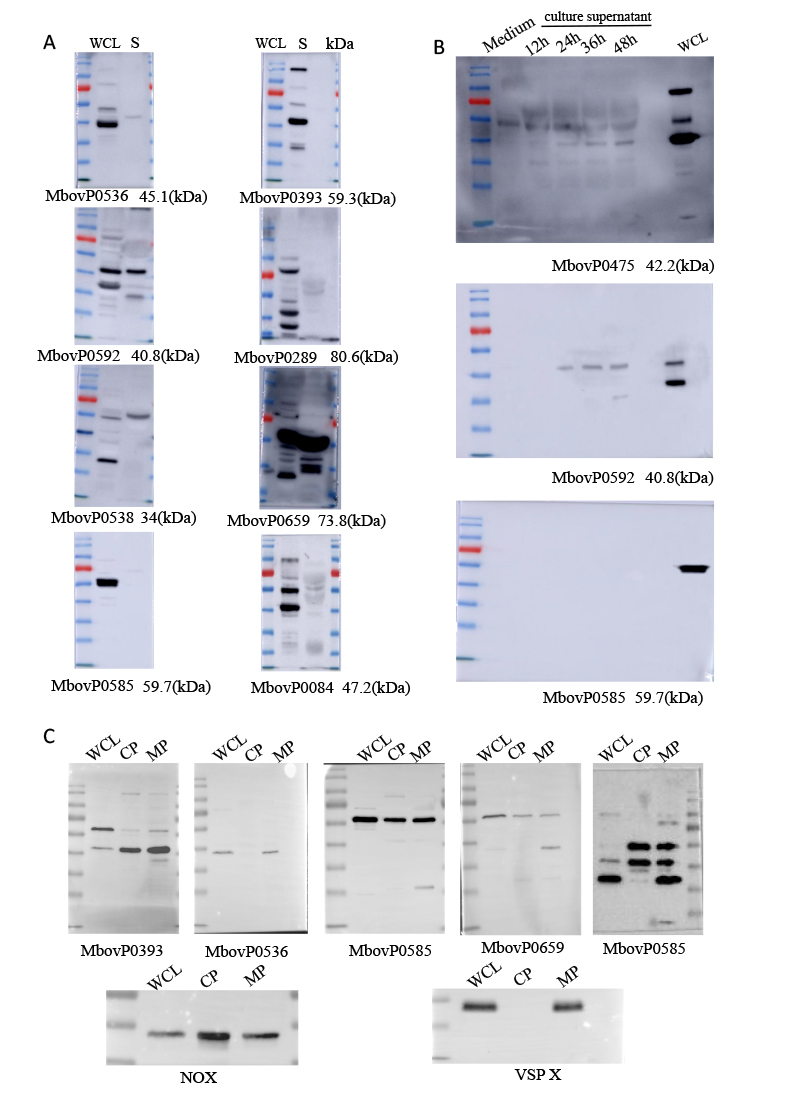
Fig. S1. Entire images of Secretory verification and subcellular localization of *M. bovis***

(A) Secretory verification of *M. bovis* proteins (entire images of Fig. 3A); (B) secretion dynamics of*M. bovis* proteins (entire images of Fig. 3B); (C)Subcellular localization of *M. bovis* proteins (entire images of Fig. 3C).
